# A Journey into the Evolution of Human Host-Oral Microbiome Relationship through Ancient Dental Calculus: A Scoping Review

**DOI:** 10.3390/microorganisms12050902

**Published:** 2024-04-30

**Authors:** Alessandra Putrino, Enrico Marinelli, Angela Galeotti, Gianmaria Fabrizio Ferrazzano, Massimiliano Ciribè, Simona Zaami

**Affiliations:** 1 Dentistry Unit, Management Innovations, Diagnostics and Clinical Pathways, Bambino Gesù Children’s Hospital, IRCCS, 00165 Rome, Italy; angela.galeotti@opbg.net (A.G.); massimiliano.ciribe@opbg.net (M.C.); 2 Department of Medico-Surgical Sciences and Biotechnologies, Sapienza University of Rome, 04100 Latina, Italy; enrico.marinelli@uniroma1.it; 3 U.N.-E.U. INTERNATIONAL RESEARCH PROJECT ON HUMAN HEALTH-ORAL HEALTH SECTION, 1200 Géneve, Switzerland; gianmariafabrizio@yahoo.it; 4 UNESCO Chair in Health Education and Sustainable Development, Dentistry Section, University of Naples “Federico II”, 80138 Naples, Italy; 5 East-Asian-Pacific International Academic Consortium; 6 Department of Anatomical, Histological, Forensic and Orthopedic Sciences, Sapienza University of Rome, 00161 Rome, Italy; simona.zaami@uniroma1.it

**Keywords:** dental calculus, palaeomicrobiology, oral microbiome, tooth decay, periodontal disease, oral health

## Abstract

One of the most promising areas of research in palaeomicrobiology is the study of the human microbiome. In particular, ancient dental calculus helps to reconstruct a substantial share of oral microbiome composition by mapping together human evolution with its state of health/oral disease. This review aims to trace microbial characteristics in ancient dental calculus to describe the evolution of the human host-oral microbiome relationship in oral health or disease in children and adults. Following the PRISMA-Extension for Scoping Reviews guidelines, the main scientific databases (PubMed, Scopus, Lilacs, Cochrane Library) have been drawn upon. Eligibility criteria were established, and all the data collected on a purpose-oriented collection form were analysed descriptively. From the initial 340 records, only 19 studies were deemed comprehensive enough for the purpose of this review. The knowledge of the composition of ancient oral microbiomes has broadened over the past few years thanks to increasingly well-performing decontamination protocols and additional analytical avenues. Above all, metagenomic sequencing, also implemented by state-of-the-art bioinformatics tools, allows for the determination of the qualitative-quantitative composition of microbial species associated with health status and caries/periodontal disease. Some microbial species, especially periodontal pathogens, do not appear to have changed in history, while others that support caries disease or oral health could be connected to human evolution through lifestyle and environmental contributing factors.

## 1. Introduction

The study of ancient microorganisms, applied to humans, helps to reconstruct the evolution of populations based on their lifestyle and environment and on contact with infectious agents and related diseases [1,2,3,4]. This assumption applies very well to the study of the human microbiome, in particular the oral one, detectable in ancient dental remains [5,6,7]. For decades, the oral health status of ancient populations has been of interest in dental archaeology and anthropology, but it was based on macroscopic observations [8]. Advancements in terms of access to genetic information about microbial communities in palaeomicrobial and palaeoenvironmental research helped to increase the quality of studies also in the subfields of archaeology and microbiology applied to oral research [9,10,11]. In fact, several moot points relative to the analysis of ancient microbial DNA, which can persist in human findings or environmental subsurfaces (deep sea, oil, permafrost), have been settled [12,13,14]. Furthermore, even if the potential of microbial research was affected by issues in experimental study design, contamination control, laboratory workflow, sequence specificity, and overall authenticity, many questions have been answered [15,16,17,18]. Research of ancient DNA requires the best possible strategy, which concerns all phases of this field of experimental study. In the last decades, studies on ancient DNA extracts revealed these samples were usually characterised by low endogenous molecule numbers as well as short and chemically altered molecules, which reduced the significance of this field of research [12,15]. As a result of these contamination issues, a number of authenticity criteria for ancient DNA sequence data have been suggested, including the use of a dedicated ancient DNA room facility (usually in a number of three) for the pre-amplification work (clean room clothing and ultraviolet C or UVC irradiation of samples and consumables; UVC light and bench for handling samples); and ancient DNA extraction and manipulation and PCR setup (all in separate hoods with internal UVC) [14,15]. In this workflow, where the access, the management of consumables and equipment, and the application of the laboratory protocol are necessarily rigorous, the development of next-generation sequencing (NGS) posed additional challenges [12]. The NGS method has contributed also to overcoming many limitations in the use of other molecular methods of pathogenic identification by enhancing investigative potential with meaningful data regarding genetic mutations, genomic rearrangement, and, more generally, the evolution of pathogens [11,12,14,19,20]. The ability to clarify eating habits, lifestyle, and health status through dental calculus, dietary microfossils, or calcified bacterial cells has been integrated through the NGS method, defining the microbiome that in ancient times contributed to the delicate balance between health and disease, as it does today [20,21,22,23,24,25]. This experimental genetic approach identifies ancient dental calculus as the preferred oral substrate because, despite the small quantities from archaeological finds, it has proved to be the best reserve of microorganisms associated with high-quality biomolecules to advance our knowledge on the bacterial/oral health status, mainly related to periodontium [26]. In fact, comparing DNA yields from paired dental calculus and dentine samples from the same tooth resulted in making dental calculus one of the richest known sources of ancient biomolecules in the archaeological record [6,26]. Even if supragingival and subgingival calculus should be collected and analysed separately, as they are known to have distinct aetiologies and different clinical significance [16], they are often difficult to distinguish in archaeological specimens. The absence of soft tissue makes it difficult to reconstruct the location of the former gingival margin, and the typical darkness or discolouration of the subgingival calculus that appears in living patients is absent in archaeological samples [23]. This condition might seem to be a disadvantage to its use for archaeological research related to oral microbiome and health/disease status in ancient populations, but since dental calculus derives from dental plaque undergone periodic and continuous mineralisation, it incorporates a great diversity of stable and well-preserved microbial and dietary microfossils (fibres, phytoliths, starch, pollen, etc.). Human life and the state of health or oral disease can be traced in dental calculus better than in other fossil substrates precisely because the calcification of the diverse bacterial biofilm structurally protects and preserves the bacterial cells from many of the biotic and abiotic factors that degrade soft tissues post-mortem [20,21,26]. Furthermore, even if both host and dietary DNA are preserved, more than 99% of the preserved DNA of ancient dental calculus is microbial in origin [4,12]. Additional sampling of associated dentine and/or bone could help just to characterize the contamination burden of the burial environment. These tissues are typically sterile during life so, bacteria recovered from them often represent post-mortem contamination and cannot be used to perform reliable research on oral microbiome addressed to describe the oral health or disease status in ancient human specimens [14,23]. The problem of environmental contamination concerns above all the comparison with the soil. It is rich in microorganisms which can form biofilms, enabling intensive inter- and intra-species interactions such as cooperation and competition like the oral environment and, consequentially, like the dental calculus [27]. Standardised collecting and sampling procedures, combined decontamination practices (UV, NaClO and EDTA treatments), adequate preservation, and sequencing, will improve the acquisition of high-quality data distinguishing host-associated microbiome from environmental contaminants [1,4,6]. Dental caries is a multifactorial non-communicable disease influenced by the host diet and arising from acid tolerating-bacteria especially from the genera *Streptococcus* (*S. mutans*, *S. sobrinus*) and *Lactobacillus*, which lead to progressive enamel demineralisation [28]. Another bacterium, a facultative anaerobe involved in various diseases, is *Streptococcus sanguinis*. The *S. mutans* and *S. sanguinis* counteract each other in the process of oral biofilm formation, as *S. sanguinis* is able to inhibit *S. mutans* development. The *Rothia dentocariosa* is a bacterium commensal aerobic and facultative anaerobic, gram-positive organism showing both coccoid and branched filament elements. It is associated with dental caries and dental plaque in periodontal patients. Other conditions that affect dental roots are instead predominantly caused by Gram-positive cocci and Gram-negative rods such as *Olsenella uli*, *Parvimonas micra* and *Pseudoramibacter alactolyticus* [29]. The *O. uli* is a Gram-positive anaerobic rod which has been recently recognised as a member of the endodontic microbial consortium of teeth with apical periodontitis. It is found in the common microbiota associated with primary endodontic infection [26]. The *P. micra* is a Gram-positive anaerobic coccus isolated from multiple polymicrobial infections such as periapical and endoperiodontal lesions, periapical abscesses and periodontitis [25], while *P. alactolyticus*, is an anaerobic Gram-positive rod considered by some authors as a good candidate for participation in the etiology of different forms of periradicular diseases. Moreover, it is amongst the most frequently identified microorganisms in the root canal of necrotic teeth associated with acute periapical abscesses [26]. In contrast, gingivitis is associated with high levels of anaerobic bacteria, especially the Gram-negative proteolytic species belonging to the genera *Fusobacterium*, *Prevotella*, *Treponema*, *Prophyromonas* and *Tannerella* (the last three known as the “red complex”) detectable in periodontal pockets [30]. The “red complex” acts synergically secreting virulence factors, leading to the inflammation of the host periodontal tissue, bone immuno-inflammatory resorption and chronic periodontitis and other forms of periodontal disease [25,26]. The current state of knowledge on oral microbiology and on the role of the microbiome in favouring or protecting the host from the onset of the most common oral diseases, such as caries and periodontitis, leads us to consider a much more varied population of microorganisms which, in the form of organised colonies, act on the state of health and disease of the oral cavity [31,32]. When the microbial composition, activity and ecology keep a balanced relationship with the host, this leads to sound oral health. In healthy adults, the majority of species belong to the bacterial phyla *Firmicutes*, *Proteobacteria*, *Actinobacteria*, *Bacteroidetes* and *Fusobacteria* [33]. Since environmental and lifestyle factors influence oral health and disease, ancient dental calculus provides an opportunity to understand the relationship between microbial patterns and oral health and disease in key transition periods in our history [34]. In order to answer the research question “what scientific findings on oral health and diseases can be found from human oral microbiome data collected on ancient dental calculus samples?”, this scoping review aimed to map the scientific evidence on oral health or disease conditions in ancient human remains of children and adults, through dental calculus samples.

## 2. Materials and Methods

The research for the articles useful for the review was last conducted on 20 November 2023 to evaluate the evidence in the scientific literature given to the collection of ancient oral microbiome data on human dental calculus specimens. Two independent experts in dentistry, forensic anthropology, and forensic science (A.P. and E.M.) consulted the English-language literature available for full-text reading on the scientific literature databases: PubMed, Scopus, Lilacs, Cochrane Library following the framework for scoping reviews of the PRISMA-ScR (PRISMA Extension for Scoping Reviews) guidelines [35]. The following eligibility criteria were set in order to select potentially relevant studies: English language, abstract and full-text reading, describing methods of genetic analysis on specimens, reporting microbial populations’ data useful to determine the influence of ancient oral microbiome on oral health or disease status in adults and children. No limit was put on the year of publication. All the study designs were considered eligible except for reviews. Studies that did not meet the eligibility criteria were discarded. Specifically, the exclusion criteria were: studies not related to the topic, written in languages other than English, without abstract and/or not available for full-text reading (also after direct request to their authors), not clearly describing the methodological genetic analysis used, studies in vitro or on animal specimens, studies not reporting microbial data composition in relation to a detected/suspected status of oral health or disease. The eligibility criteria above described implied the aim of satisfying the search strategy also with respect to achieving the main quality data: availability, usability, reliability, relevance and readability. The sources search has been performed using “AND” and “OR” Boolean operators between Mesh terms or free text terms combined as follows:

1. Human Microbiome
2. Dental Calculus
3. Tooth
4. Oral health
5. Oral disease
6. 1 OR 2 OR 3 OR 4 OR 5
7. DNA, Ancient
8. Metagenomics
9. High-Throughput Nucleotide Sequencing
10. Biological Evolution
11. 7 OR 8 Or 9 OR 10 OR 11
12. 6 and 11

The results were unified and compared using the Zotero bibliographic software (Zotero 6 for Windows, Corporation for Digital Scholarship, Vienna, VA, USA) to delete duplicate results. A third operator expert in forensic science and forensic anthropology (S.Z.) supervised the research of the articles to verify that the articles selected met the standards set for the scoping review. The inclusion of possible doubtful articles was discussed. References to the selected articles and the discarded reviews were consulted to avoid overlooking additional potentially valuable sources. The contents of any included studies were charted according to the authors and year of publication, geographical origin and historical period of dental calculus specimens, sample size (if applicable), genomic analysis method, and oral microbiome population. Subsequently, these results were analysed and discussed in this article. The scoping review search strategy was depicted as a flow diagram using the PRISMA model for systematic reviews [36].

## 3. Results

**Figure 1:**
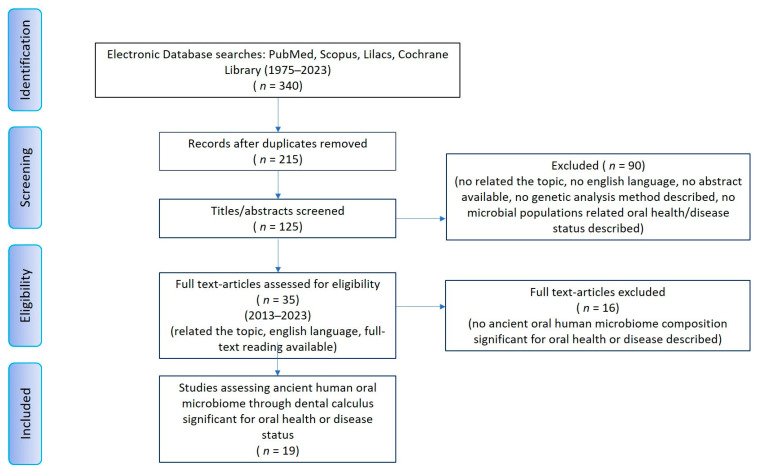
The PRISMA flow diagram with the search strategy used in this scoping review.

Initial results on the reference databases led to the identification of 340 scientific papers of potential relevance (published between 1975 and 2023). After the removal of duplicated results from the different databases (*n* = 215), 125 articles were screened in detail. The articles eligible for full-text review were ultimately 35. After perusing the full-text versions, 19 articles were selected for the purpose of framing the scoping review (Table 1). They were published between 2013 and 2023 [7,21,22,24,37,38,39,40,41,42,43,44,45,46,47,48,49,50]. No data emerged about the age of the ancient population sampled, except for one study reporting data on a juvenile mandible [47], and others clarified that the ancient population was of adult age [46,49]. The flow diagram (Figure 1) describes the entire search strategy and review process.

### 3.1. Geographical Origin and Historical Dating of Dental Calculus Samples

The human dental remains on which the dental calculus samples used in the studies were found come from archaeological sites and cemeteries in various countries; European countries are the most represented. There are studies with samples taken from the Balkans, Belgium, Denmark, France, Germany, Italy, Latvia, the Netherlands, Poland, Spain, and the United Kingdom [7,24,26,37,38,39,40,43,44,45,46,47,49,50]. Non-European countries are represented by only two studies: Egypt and Israel [42,47]. Some studies use previously published datasets from samples from different parts of the world (labelled as “miscellaneous worldwide”) [21,22]. A study does not specify the samples’ geographical origin [40]. The human remains herein taken into account range from 6000 years ago to the 19th century [7,21,22,24,26,37,38,39,40,41,42,43,44,45,46,47,48,49,50].

### 3.2. Sample Size and Site of Dental Calculus Collection

The sample size in several studies is made up of the number of unit samples of dental calculus analysed [7,21,22,38,39,40,42]. In fact, in the studies herein included, the sample size often refers to the number of subjects from whose remains the sample was taken, without specifying the number of samples per oral or dental site on the same individual [24,37,38,39,43,44,45,46,47,48,49,50]. A study does not report any detail about this information [41]. In addition, dental calculus origin, i.e., from which teeth the sample was taken or from which tooth surfaces, is not always specified [22,37,46]. 

### 3.3. DNA Sequencing Technology

Most of the studies have used shotgun metagenomic sequencing analysis as a method of studying the microbial composition of the dental calculus sample for comparative purposes with samples from other eras or taken from modern populations. Two studies report different techniques, namely the extraction of bacterial DNA and PCR amplicon libraries of the 16s rRNA gene [37] and scanning electron microscopy (SEM) analysis [47]. Some studies also rely on the description of additional analyses such as Random Forest Analysis [37], Metaproteomic Analyses [39,48], and Bioinformatics Tools [21]. Decontamination protocols are sometimes described as one of the main purposes of the study itself [43]. 

### 3.4. Bacterial Composition Dominance in Ancient Dental Calculus

Most studies highlight the presence of periodontal pathogenic bacteria comparable to modern dental calculus specimens: *Porphyromonas gingivalis*, *Tannerella forsythia*, *Treponema* spp. [7,21,22,26,37,38,39,40,42,44,48,49,50], *Actinomyces* spp., *Olsenella*, *Fretibacterium*, *Desulfobulbus* Oral Taxon 041 [7,22,24,26,39,40,42,44,45,48,49,50], *Streptomyces* spp. [22]. *Methanobrevibacter oralis*, an archaeal genus believed to be an important periodontal disease pathogen, has been described in many studies [22,38,40,44,46,48,50]. *Saccharibacteria/TM7* phylum is present in the dental calculus of ancient adult skeletons with evidence of mild to severe periodontal disease [41]. *Cocci* and *filamentous bacteria*, associated with modern mild juvenile periodontitis, were detected in ancient samples as well [47]. Other studies also highlight the presence of bacteria more related to caries disease: *Veillonellaceae* [37,48] and *Rothia dentocariosa* [26]. Bacteria typically associated with modern tooth decay are not chronologically predominant in older populations: *Streptococcus mutans* and *Sobrinus* [21,37,38,43,45,48,49]. *Aggregatibacter* and *Neisseria* present in ancient dental calculus specimens document oral health status [48].

**Table 1:** Articles included in the Review listed by year of publication and reporting geographic origin and time period of the ancient dental calculus sampled, sample source and size, analysis method and bacterial composition.

Authors—Year	Geographical Origin	Time Period	Sample Size and Site	DNA Sequencing Technology	Bacterial Composition Dominant in Collected Ancient Dental Calculus Samples
Adler et al., 2013 [37]	Poland	From 10th–6th BCE to 500–1500 CE	Supra and subgingival dental calculus from 34 human skeletons	Extraction of bacterial DNA and generation of PCR amplicon libraries of the 16S rRNA gene; Random Forest analysis	*Proteobacteria*, *Firmicutes*, *Actinobacteria*. *Veillonellaceae* (tooth decay), *P. gingivalis*, *Tannerella*, *Treponema* (p. disease). *S. mutans* is not dominant.
Warinner et al., 2014 [49]	Germany	From 500 to 1500 CE	Dental tissues of 4 four adult human skeletons	Meta-genomic-shotgun sequencing and 16s rRNA amplicon	*Tannerella forsythia*, *Porphyromonas gingivalis*, and *Treponema denticola* are particularly abundant. Additional pathogens include those implicated in acute dental infections (e.g., *Actinomyces odontolyticus*) and caries (*S. mutans*). *Filifactor alocis* and *Olsenella uli* have recently been associated with periodontitis and endodontic infections, respectively.
Weyrich et al., 2017 [38]	Belgium, Italy, Spain	From 12th to 10th millennium BCE	Dental calculus (24 specimens) from 5 skeletons	Metagenomic-shotgun sequencing and 16s rRNA amplicon (v4 region)	*S. mutans* presence is irrelevant. *P. gingivalis*, *T. forsythia*, *T. denticola* are abundant. Abundance of *Methanobrevibacter oralis*.
Jersie-Christensen et al., 2018 [39]	Denmark	1100–1450 CE	Dental calculus (22 specimens) from 21 human remains	Metaproteomic and metagenomic analysis	3671 protein groups, covering 220 bacterial species and 81 genera across all medieval samples. After *Actinomyces* spp., the genera *Olsenella* and *Fretibacterium*, both of which have been implicated in periodontitis, are the most abundant. Significant contributions from *Fretibacterium* spp., *Porphyromonas* spp., *Treponema* spp., *Tannerella* spp., and *Desulfobulbus* sp. oral taxon 041; all of which have been suggested to be involved in clinical periodontitis.
Mann et al., 2018 [22]	Miscellaneous worldwide	500–1500 CE	48 specimens of dental calculus and dentin	Metagenomic- shotgun sequencing	*Methanobrevibacter*; *Tannerella*; *Porphyromonas*; *Actinomyces*; *Streptomyces*
Willman et al., 2018 [26]	France	18th century	Specimens from 9 subjects	High-Throughput DNA sequencing (HTS)	The presence of *Streptococcus mutans* and also *Rothia dentocariosa*, *Actinomyces viscosus*, *Porphyromonas gingivalis*, *Tannerella forsythia*, *Pseudoramibacter alactolyticus*, *Olsenella uli* and *Parvimonas micra* was confirmed like specific bacterial signature associated to carious or periodontal pathologies.
Velsko et al., 2019 [40]	United Kingdom	1778–1785	48 samples of historic dental calculus	Metagenomic-shotgun sequencing	Many of the taxa with higher abundance in calculus are “late colonizers” (i.e., *Desulfobulbus*, *Methanobrevibacter*, *Tannerella*); *P. gingivalis* and *T. forsythia* characterised historic periodontal disease site from historic healthy site calculus as they do in modern plaque.
Achtman et al., 2020 [21]	Miscellaneous worldwide	Not specified	110 samples of ancient dental calculus	Meta-genomic-shotgun sequencing data sets analysed with new bioinformatic tools (SPARSE, EToKi, GrapeTree)	*Streptococcus sanguinis* and *Tannerella forsythia* were most abundant in historical dental calculus (periodontal disease). *Treponema denticola* was most frequently found in historical dental calculus but *Porphyromonas gingivalis* is most frequent in modern plaque and is generally much less abundant.
McLean et al., 2020 [41]	Not Specified	From 6th–10th BCE to 500–1500 CE and 19th century CE	Not Specified	Metagenomic-shotgun sequencing	*Saccharibacteria/TM7 phylum* is present (G1 group genome) in dental calculus of ancient adult skeletons with evidence of mild to severe periodontal diseases.
Neukamm et al., 2020 [42]	Egypt	From 2196 BCE to 395 CE	5 ancient dental calculus samples	Metagenomic-shotgun sequencing	Red Complex bacteria (*Tannerella forsythia*, Porphyromonas gingivalis, and *Treponema denticola*); Two other bacteria (*Filifactor alocis* and *Olsenella uli*) associated with periodontitis and endodontic infections were also identified with damage profiles. In general, the calculus samples are dominated by *Firmicutes*, *Actinobacteria*, *Proteobacteria*, *Bacteroidetes*, *Chloroflexi*, *Fusobacteria*, and *Spirochetes.*
Farrer et al., 2021 [43]	United Kingdom	1170–1290 CE	Dental calculus samples extracted by 26 individuals buried	Metagenomic-shotgun sequencing and decontamination protocols	Three oral species—*Actinomyces* sp., *Olsenella* sp. and *Streptococcus sanguinis* were more likely to be present in the EDTA and UV + NaClO groups than the others.
Granehäll et al., 2021 [44]	Italy	6000–3500 BCE to 400–1000 CE	Dental calculus from 20 ancient human skeletal remains	Metagenomic-shotgun sequencing	Red complex and besides the presence of *M. oralis*
Kazarina et al., 2021 [7]	Latvia	16–17th century AD	15 historic dental calculus samples	Metagenomic-shotgun sequencing	Historic dental calculus samples represented a slightly different pattern of the most abundant species, the first 10 of which were *Olsenella* sp. oral taxon 807, *Actinomyces* sp. oral taxon 414, *Anaerolineaceae bacterium* oral taxon 439, *Pseudopropionibacterium propionicum*, *Streptococcus sanguinis*, *Eubacterium minutum*, *Desulfobulbus oralis*, *Lautropia mirabilis*, *Streptococcus cristatus*, and *Ottowia* sp. oral taxon 894.
Ottoni et al., 2021 [45]	Balkans and Italy	From 3rd BCE to 500–1500 CE	Dental Calculus from 44 prehistoric foragers and farmers	Metagenomic-shotgun sequencing	Prevalence of *Anaerolineaceae bacterium* oral taxon 439, *M. oralis*, *Desulfomicrobium orale*, and *Desulfobulbus oralis*. Neolithic farmers possessed a higher frequency *Olsenella* sp. oral taxon 807. The analysis of differential species abundances showed that *Olsenella* sp. oral taxon 807 and *Anaerolineaceae bacterium* oral taxon 439 were more abundant in the Neolithic farmers, whereas *Streptococcus sanguinis* was higher in the Mesolithic foragers.
Fagernäs et al., 2022 [46]	Spain	4500–5000 BP	Dental calculus from 4 adults’ skeletons with Brothwell score from 1 (slight) to 4 (gross);	Metagenomic-shotgun sequencing; A total of two separate analyses were conducted, one without occlusal samples and one including occlusal samples.	*Methanobrevibacter* and *Olsenella*
Garralda et al., 2022 [47]	France, Israel	75–70/60 ky BP	Dental calculus from the left corpus of a juvenile mandible (France) compared with dental calculus of 2 adult Neanderthals from different locations (Israel)	SEM analysis	*Cocci* and filamentous types of bacteria in the French sample; more *filamentous bacteria* are present in the Israeli samples.
Scorrano et al., 2022 [48]	Italy	50–12 ky BP	Dental Calculus from the remains of 2 hunter-gatherers (San Teodoro 3 and San Teodoro 5)	Metagenomic-shotgun sequencing	Abundance in *Actinomyces*, *Streptococcus* and *Propionibacterium*, genera typically associated with the oral microbiome. In one sample abundant *Olsenella* (known to cause endodontic infections) while in the other was abundant *Aggregatibacter* and *Neisseria*, (normal oral microbiome). Most of the ancient calculus samples were also highly abundant in the periodontal component: *Prevotella conceptionensis*, *Porphyromonas gingivalis* and *Prevotella nigrescens*. Found species including *Methanobrevibacter oralis* and *Olsenella* sp. Oral taxon 807 among those with significantly higher abundance in the ancient samples. *Porphyromonas gingivalis* and *Treponema denticola* were more abundant in samples with periodontal disease and ancient calculus.
Velsko et al., 2022 [24]	Netherlands	19th Century	Dental calculus from 75 skeletal collections	Metagenomic-shotgun sequencing	The top species with strongest negative loadings are largely anaerobic taxa that are dominant in mature oral biofilms, including those in the genera *Methanobrevibacter*, *Eubacterium*, *Desulfobulbus*, *Fretibacterium*, and *Tannerella*.
Gancz et al., 2023 [50]	United Kingdom	2200 BCE to 1853 CE	235 ancient dental calculus samples	Meta-genomic-shotgun sequencing	*Methanobrevibacter* was not common in the oral microbiome of modern industrialised societies. Its disappearance suggests pre-industrialised microbiomes were more diverse than previously recognised, enhancing our understanding of chronic, non-communicable disease origins in industrialised populations.

## 4. Discussion

### 4.1. Dental Calculus and Oral Health and Disease

Dental calculus is a calcified dental plaque in which inorganic (mineral salts) and organic components (proteins and carbohydrates) are present. In fact, it is primarily made up of calcium phosphate mineral salts deposited between and within the remnants of formerly viable microorganisms [6]. A viable dental plaque covers mineralised calculus deposits [18]. According to its location, it can be divided into supragingival and subgingival calculus. Its presence contributes to periodontal disease [20]. Levels of supragingival calculus can be successfully kept under control with proper oral hygiene, regular professional care, and sound eating habits [51]. Although susceptibility to periodontal disease and caries is strongly linked to the subject’s habits, the build-up of dental plaque and dental calculus, both supra- and subgingival, is a risk factor [52]. Moreover, the composition of the oral microbiome with specific signatures can significantly modulate the state of oral health or disease by interacting with the host [53]. For this reason, and because of the great importance that oral microbiome has at a systemic level as well, modern medicine and dentistry are trying to shed light on such an influence and the mechanisms related to the microbiome-host interaction [54]. While nowadays dental calculus is routinely removed to maintain oral health, its value as an informative element to understand ancient diet and health has been acknowledged [55]. The data and findings herein accounted for have highlighted the composition of the oral microbiome extracted from the dental calculus in ancient human remains, either in teeth with carious and/or periodontal lesions or healthy teeth. Such correlations have clarified the differences with the modern microbiome under the same conditions and thus traced a possible relationship in human evolution. Modern oral hygiene practices that disrupt natural oral biofilm development and maturation may be responsible for the major differences observed in many studies comparing modern dental calculus samples [24,40,41]. Some studies also assert that the ancient (middle age) oral microbiome was more heterogeneous than the modern one [39]. The definition of the state of oral health or disease, mostly understood as the carious or periodontal lesion, is not always a datum supported by the description of the bone or dental profile of the teeth on which the dental calculus sample was taken. Some authors found no difference between ancient calculus from the molars and anterior teeth of the samples, indicating that the sampling site would not affect the composition [45]. Other data, on the other hand, support the importance of the site from which the sample of dental calculus is taken. Occlusal calculus, for example, was found to have a higher amount of DNA damage than other calculus [46]. According to current knowledge, the quality and quantity of dental calculus in living humans are affected by the position of the salivary glands in relation to the ducts, and this is an important aspect that often cannot be taken into account in archaeological studies [54,55]. However, more recent data on ancient dental calculus support the evidence that even in macroscopically healthy teeth, oral microbial characteristics typical of carious or periodontal lesions were present, thus defining the state of health or oral disease of a subject whose teeth have not come down to us over time. Even if the age of the ancient population in the studies selected is not reported except for a few results [46,47,49] we should consider that the attainment of adulthood has gradually been prolonged over the course of human evolution, and therefore the population to which studies on very ancient samples refer probably includes a population with an age close to adolescence.

### 4.2. Modern and Ancient Oral Microbiome

In ancient dental calculus, as also observed in modern specimens, differences were found in taxa between anterior (incisors and canines) and posterior (premolars and molars) teeth, where the taxa that are more abundant in the anterior teeth are more often aerobic or facultatively anaerobic, while the taxa that are most associated with posterior teeth are anaerobic [56]. *Actinomyces*, *Streptococcus*, *Propionibacterium*, *Aggregatibacter* and *Neisseria* are present in ancient healthy oral microbiomes [24,48]. However, the high incidence of samples on teeth with caries or periodontal lesions makes this evidence, partly overlapping with modern oral health, less significant than pathogenic microbial composition [10,28,33].

Ancient dental calculus in interproximal spaces seems to be enriched in species belonging to the genera *Methanobrevibacter* and *Olsenella*, which are both acid-tolerant anaerobes [7,22,24,38,40,43,44,46,48]. In the modern era, *Methanobrevibacter* is often associated with severe periodontitis and peri-implantitis, but its pathogenic roles remain unclear [57,58]. *Olsenella* is typically associated with symptomatic endodontic infections and periodontal-disease-associated biofilm, both in modern times and ancient finds [26,49,59,60].

As for periodontal health or disease status, among the studies included in this review, relatively few, for instance, refer to the Brothwell classification (to describe bone damage from periodontal disease) with a score from 1 (slight) to 4 (gross) [39,44,45,46]. Several different studies have found the ‘red complex’ members *Treponema denticola*, *Tannerella forsythia*, *Porphyromonas gingivalis*, but also *Desulfobulbus* (late colonisers of oral plaque), *Anaerolineaceae bacterium* oral taxon 43, *Fretibacterium*, *Filifactor*, in deep periodontal pockets [7,24,26,39,40,42,44,45,49].

They could indicate a difference in the level of maturation and, possibly, disease state (severity of periodontitis) similar to modern human beings [44]. Dental calculus’ microbiome analysis has identified ‘red complex’ bacteria correlated with periodontal diseases even in Ancient Egypt [42].

Some authors also report a probably self-treated dental abscess in the Late Pleistocene based on plant bacteria found in the sampled dental calculus as a sign of human evolution rather than as a unique system useful for the study of long-term microbial evolution [38]. The SEM analysis of Neanderthal dental calculus demonstrated the prevalence of *coccitype* bacteria, quite common in juvenile individuals with mild periodontitis, in the skeletal remains of an esteemed young male. This group of microbiotas was less prevalent in another Neanderthal adult male, where “*rods*” were more frequent, although less so than in other older adults.

These macromorphological studies on dental calculus samples reflect the development of the microbiota related to the different diets and ages at the death of these Neanderthals, evolving through life to include more rods, as is known for modern populations [47].

According to less recent studies, the composition of oral microbiota has remained surprisingly constant between Neolithic and Medieval times [7,37], after which (the now ubiquitous) cariogenic bacteria (*Streptococcus mutans*) became dominant, apparently during the Industrial Revolution.

Modern oral microbiota is markedly less diverse than the one in historic populations, which might be contributing to chronic oral (and other) diseases in the post-industrial age [36]. The most significant evolutionary changes in the human diet involve the adoption of carbohydrate-rich foods (Neolithic Age) in the epochal transition from hunter to farmer lifestyle and later the advent of industrially processed flour and sugar [37]. Such conclusions are supported by other authors as well, who found no significant differences in the taxonomic composition of the historic dental calculus samples classified by lifestyle (town and countryside), tooth type and presence/absence of caries [7]. In Italy, the oral microbiome of the Palaeolithic and Mesolithic foragers was not significantly different from the one found in Neolithic farmers [45]. When drawing distinctions among the chronological groups of this study from a geographical standpoint, the authors found significant differences between the foragers from Italy and the Danube Gorges, which may point to geographic structuring in the oral microbiome composition [45]. As for most archaeological samples from the Medieval period, it is worth remarking that their microbial composition in dental calculus will be more similar to the modern samples, especially if they belong to individuals who possibly had better access to foods and higher social status (e.g., dietary carbohydrates such as finely ground bread, sweets, and meat) [7,37]. However, in other studies accounting for samples from the 19th century, the subjects exhibited relatively poor oral health with high rates of periodontal disease, caries, heavy calculus deposits, and antemortem tooth loss. In this case, no associations were found between pipe notches and dental diseases, or microbial species composition.

Calculus samples before and after the introduction of tobacco showed highly similar species profiles [34]. The most abundant bacterial taxa detected in many historical calculus samples included several commensal bacterial species commonly found in the human oral cavity, such as *Streptococcus sanguinis*, *Streptococcus cristatus*, and Lautropia *mirabilis* [7,37]. Other authors, on the contrary, point out that *Streptococcus mutans* and *Streptococcus sobrinus* (associated with dental caries) were rare in historical dental calculus but common in modern plaque and even more common in saliva [41]. The periodontal pathobiont *Desulfobulbus oralis*, known for its ability to trigger a proinflammatory response in the oral epithelium, was one of the most abundant bacterial species in postmedieval dental calculus samples [7]. In the Middle Ages, the *Desulfomicrobium orale*, absent in modern samples and at very low abundance in healthy ancient specimens, is another emerging pathogen of interest [39]. Key adaptations within different *Saccharibacteria* groups may have occurred during the transition from the environment to the eventual survival and persistence within human hosts. Multiple lines of evidence now strongly confirm that all major groups of *Saccharibacteria* are highly prevalent in humans and should be considered part of the core oral microbiome [41]. Many results are consistent and point to the relative stability in the composition of the oral microbiome at definite time points [37,40]. According to other theories, dental calculus may not preserve microbial indicators of health and disease status as distinctly as dental plaque. This indicates that calculus microbiomes are primarily shaped by individual-specific biofilm developmental processes that are independent of host health [24]. However, several virulence factors, fimbriae, identified from ancient species have higher expression than in the equivalent modern types, and they could work as critical mediators of initial adhesion and for the invasion of host cells, and together with gingipains, they could play several roles in pathogenicity [39,44].

### 4.3. Genomic Analyses on Modern and Ancient Dental Calculus

Genomic analyses of modern dental calculus are less problematic than those of historical dental calculus findings. Strict protocols for the extraction and amplification of microbial or host DNA, also relying on decontamination procedures to isolate the most precise information possible from very small fragments of interest, are essential for that purpose [16,21,22,39,45,46,56]. In fact, it is not uncommon for the results to include data relating to environmental microbial species, characteristic of the soil where the skeleton was found, or other species linked to the degradation process of the corpse, therefore unrelated to the host’s microbial profiling or their microbiome [56,57]. Furthermore, the authenticity of the historical microbiome data need to be confirmed using DNA damage patterns. In fact, the rate of deamination increases over time, and the characteristics of damaged DNA patterns can confirm the origin of the DNA [48,56,61]. The recording and sharing of genomic data sets from most of the published studies and the analysis of modern DNA microbiome reserves databases have made it possible to amplify and update many results over time [21,22,57,62]. Such an approach is made possible by the availability and development of increasingly high-performance bioinformatics tools [43]. The fundamental assumption, which has emerged and been recommended by a few studies, is that, given the smallness and non-repeatability of the sample, operators who handle the finds for the first time since their discovery must be very careful in their collection and conservation [7,21,24,44].

Many of the dental calculus samples have a predominant dental calculus microbial signature indicating sufficient preservation of the oral microbiome in a mineralised oral plaque biofilm. 

The impact of laboratory and environmental contamination on the study samples, which is usually quite low, can be assessed by additional analyses [7,21]. In fact, many studies can identify and evaluate contamination of bacterial populations linked to soil, or arising from body degradation processes or plant substrates, all unrelated to the composition of the oral microbiome and dietary/lifestyle aspects [24,46,48]. Relevant evidence points to higher levels of oral microbiome in dental calculus samples, which are different and less contaminated than those found in dentin. One-third of teeth should retain DNA from the oral microbiome, and thus dentin could serve as an alternative source of oral bacterial DNA only in the absence of preserved dental calculus deposits [22]. Dental calculus can therefore provide a favourable environment for long-term DNA preservation and a valuable source of information as to diets based on meat or fish, vegetables, or mushrooms [38,48]. The opportunity to directly study related biomolecules and microfossils incorporated in the calculus during an individual’s lifetime, for example, plant DNA, stresses the importance of including environmental controls or additional lines of biomolecular evidence in dietary interpretations in order to rule in or out the presence of non-host DNA of postmortem origin [46]. Such a level of precision can go a long way towards shedding light on the ancient oral microbiome as a trace of human evolution and on the connections that the balance or imbalance of the host-microbiome system may have on the evolution of the state of oral health and disease [38]. By studying host–microbiome coevolution with a hologenomic or multi-omic approach, integrating osteological dental health data in archaeological populations with ancient metagenomics could offer unique insights into health and disease processes and could help to establish that the strongest factors shaping microbiome composition in living populations differ from that functioning on evolutionary timescales [24,40,48]. Proof of this is provided by a study that analyses the dental calculus of specimens from the Mesolithic era to the Middle Ages, focused on the less documented type of bacteria, *Saccharibacteria* [41]. Furthermore, quantitative metaproteomics has the potential to provide additional levels of molecular information about the oral health status of individuals in archaeological contexts [39].

## 5. Conclusions

Currently, the available research findings point to the ability of ancient dental calculus, when appropriately collected and processed for decontamination, to provide very significant information on the composition of the oral microbiome from antiquity to the present. Metagenomic analyses or bioinformatics tools that expand information on already-analysed data sets are greatly valuable to that end. Current data support the fact that some periodontal pathogenic bacteria, such as the “red complex”, typical of modern periodontal disease, have always been present in the human oral microbiome, regardless of age, eating habits, or geographical/environmental factors.

The prevalence of periodontal disease in human evolutionary history is not easily evaluable because clinical metrics are difficult to apply to ancient specimens. However, the high abundance of periodontal pathogens taxa (*P. gingivalis*, *Tannerella forsythia*, *Treponema denticola*) and host innate immune proteins found in ancient dental calculus specimens strongly suggest periodontitis-associated inflammation. The link between periodontitis and modern diseases, i.e., cardiovascular diseases and stroke, is extremely significant since archaeological evidence confirmed the high prevalence of atherosclerosis since prehistory.

Dental caries are easily observed in the archaeological record. Extensive data have now been collected on ancient populations, spanning time periods from early hominin to populations lived in the 19th century. It is clear from these studies that diet is the major driver of caries frequency. This origin is similar to the modern’s extreme levels of dental decay. Oral hygiene regimens and prophylactic dental care became necessary measures to prevent premature tooth loss. Although bacteria linked to modern caries disease have always been present, especially *S. mutans*, they have become more prevalent along with the growing consumption of refined carbohydrates, such as flour and sugar, especially sucrose. However, it is still not clear in what degree the oral microbiota of the populations changed in step with subsistence practices. Even if reconstructing the full polymicrobial community contributing to ancient carious lesions is challenging, further ancient DNA investigations of dental calculus are desirable to reveal the natural history of *S. mutans* and determine the major issues of its evolution and role within the human oral cavity.

Bacterial species that are less evident in modern dental calculus specimens are emerging in ancient specimens, but their significance in the evolution of the composition of the oral microbiome needs to be investigated by further studies. The significant absence of bacteria that maintain a state of eubiosis with the host is a distinctive feature of ancient samples, which points to the precariousness of oral health, whose importance has been greatly acknowledged in modern times. Current evidence does not allow us to trace an evolution of the host-microbiome relationship based on the age range of the ancient populations from which dental calculus samples were taken.

Considering that life expectancies throughout history have gradually increased with civilisation, an interesting approach would be to profile the ancient population object of these studies by presumed age. There is still a need to investigate this branch of palaeomicrobiology, since the heterogeneity of ancient oral microbiome composition, compared to the modern one, can still provide key answers on human evolution. Further knowledge of the ancestral oral microbiome can be leveraged to improve human health today.

## Data Availability

Not applicable.
